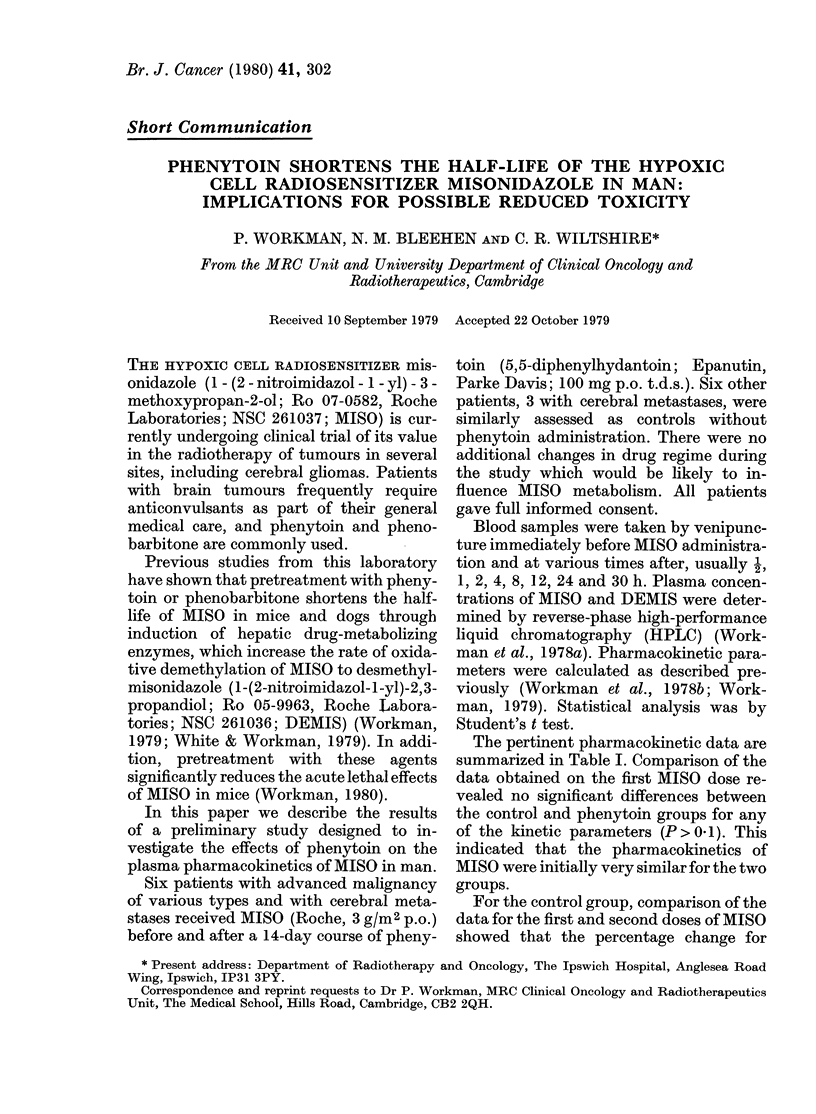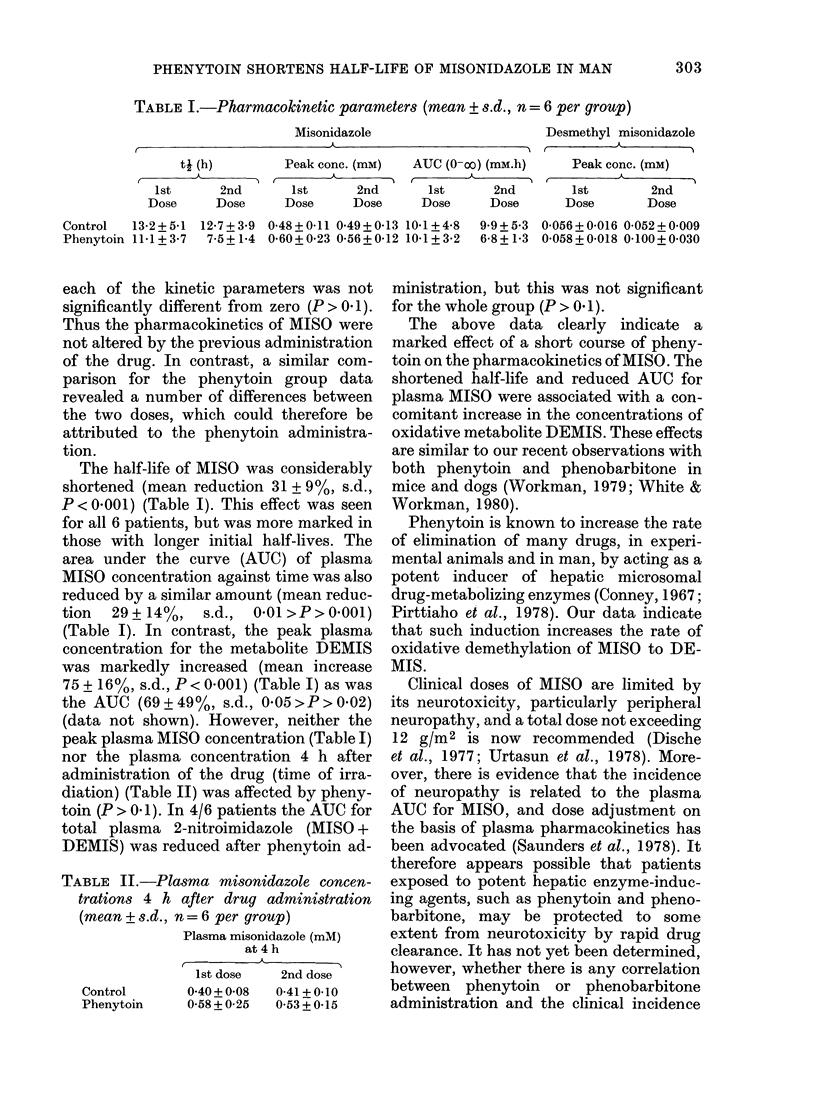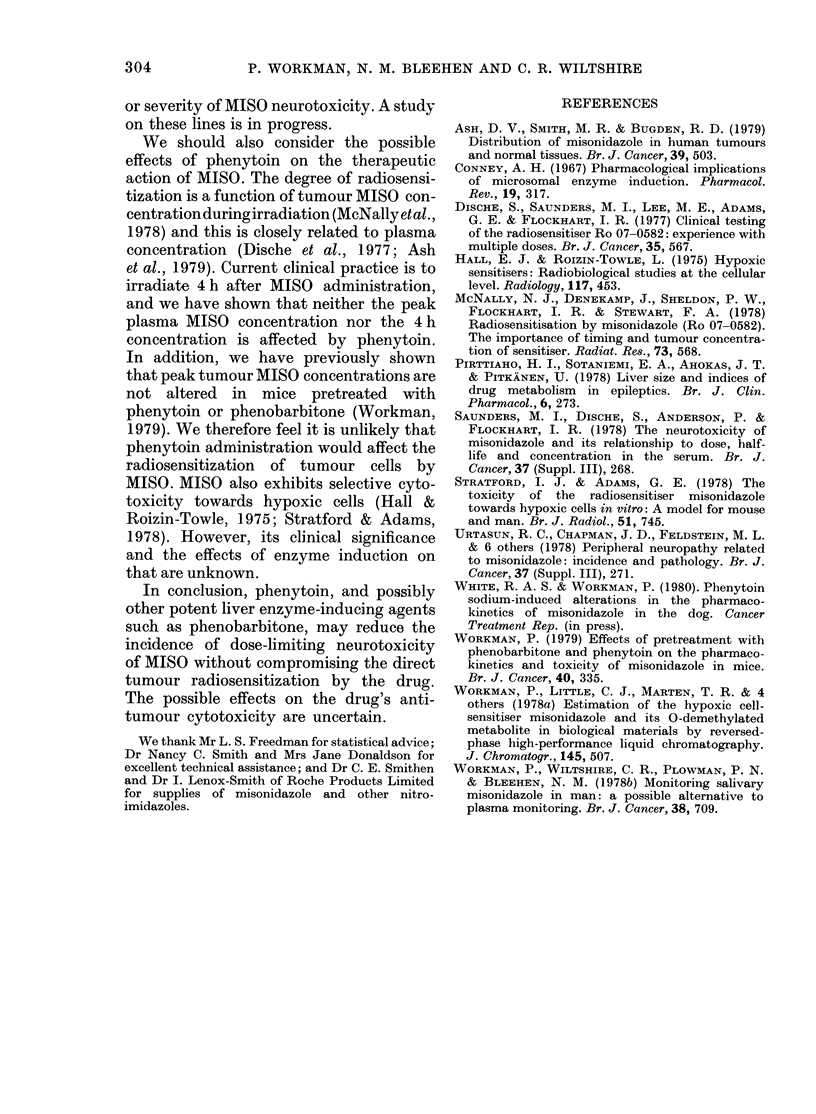# Phenytoin shortens the half-life of the hypoxic cell radiosensitizer misonidazole in man: implications for possible reduced toxicity.

**DOI:** 10.1038/bjc.1980.43

**Published:** 1980-02

**Authors:** P. Workman, N. M. Bleehen, C. R. Wiltshire


*Br. J. Cancer* (1980) **41**, 302

***Short Communication***

### PHENYTOIN SHORTENS THE HALF-LIFE OF THE HYPOXIC CELL RADIOSENSITIZER MISONIDAZOLE IN MAN: IMPLICATIONS FOR POSSIBLE REDUCED TOXICITY

P. WORKMAN, N. M. BLEEHEN AND C. R. WILTSHIRE*

*From the MRC Unit and University Department of Clinical Oncology and Radiotherapeutics, Cambridge*

Received 10 September 1979 Accepted 22 October 1979

THE HYPOXIC CELL RADIOSENSITIZER misonidazole (1 - (2 - nitroimidazol - 1 - yl) - 3 - methoxypropan-2-ol; Ro 07-0582, Roche Laboratories; NSC 261037; MISO) is currently undergoing clinical trial of its value in the radiotherapy of tumours in several sites, including cerebral gliomas. Patients with brain tumours frequently require anticonvulsants as part of their general medical care, and phenytoin and phenobarbitone are commonly used.

Previous studies from this laboratory have shown that pretreatment with phenytoin or phenobarbitone shortens the half-life of MISO in mice and dogs through induction of hepatic drug-metabolizing enzymes, which increase the rate of oxidative demethylation of MISO to desmethylmisonidazole (1-(2-nitroimidazol-1-yl)-2,3-propandiol; Ro 05-9963, Roche Laboratories; NSC 261036; DEMIS) (Workman, 1979; White & Workman, 1979). In addition, pretreatment with these agents significantly reduces the acute lethal effects of MISO in mice (Workman, 1980).

In this paper we describe the results of a preliminary study designed to investigate the effects of phenytoin on the plasma pharmacokinetics of MISO in man.

Six patients with advanced malignancy of various types and with cerebral metastases received MISO (Roche, 3 g/m$^2$ p.o.) before and after a 14-day course of phenytoin (5,5-diphenylhydantoin; Epanutin, Parke Davis; 100 mg p.o. t.d.s.). Six other patients, 3 with cerebral metastases, were similarly assessed as controls without phenytoin administration. There were no additional changes in drug regime during the study which would be likely to influence MISO metabolism. All patients gave full informed consent.

Blood samples were taken by venipuncture immediately before MISO administration and at various times after, usually $\frac{1}{2}$, 1, 2, 4, 8, 12, 24 and 30 h. Plasma concentrations of MISO and DEMIS were determined by reverse-phase high-performance liquid chromatography (HPLC) (Workman *et al.*, 1978*a*). Pharmacokinetic parameters were calculated as described previously (Workman *et al.*, 1978*b*; Workman, 1979). Statistical analysis was by Student's *t* test.

The pertinent pharmacokinetic data are summarized in Table I. Comparison of the data obtained on the first MISO dose revealed no significant differences between the control and phenytoin groups for any of the kinetic parameters ($P > 0{\cdot}1$). This indicated that the pharmacokinetics of MISO were initially very similar for the two groups.

For the control group, comparison of the data for the first and second doses of MISO showed that the percentage change for

* Present address: Department of Radiotherapy and Oncology, The Ipswich Hospital, Anglesea Road Wing, Ipswich, IP31 3PY.

Correspondence and reprint requests to Dr P. Workman, MRC Clinical Oncology and Radiotherapeutics Unit, The Medical School, Hills Road, Cambridge, CB2 2QH.

TABLE I.—*Pharmacokinetic parameters (mean ± s.d., n = 6 per group)*

| | Misonidazole | | | | | | Desmethyl misonidazole | |
|---|---|---|---|---|---|---|---|---|
| | $t_{\frac{1}{2}}$ (h) | | Peak conc. (mM) | | AUC (0–∞) (mM.h) | | Peak conc. (mM) | |
| | 1st Dose | 2nd Dose | 1st Dose | 2nd Dose | 1st Dose | 2nd Dose | 1st Dose | 2nd Dose |
| Control | 13·2 ± 5·1 | 12·7 ± 3·9 | 0·48 ± 0·11 | 0·49 ± 0·13 | 10·1 ± 4·8 | 9·9 ± 5·3 | 0·056 ± 0·016 | 0·052 ± 0·009 |
| Phenytoin | 11·1 ± 3·7 | 7·5 ± 1·4 | 0·60 ± 0·23 | 0·56 ± 0·12 | 10·1 ± 3·2 | 6·8 ± 1·3 | 0·058 ± 0·018 | 0·100 ± 0·030 |

each of the kinetic parameters was not significantly different from zero ($P > 0·1$). Thus the pharmacokinetics of MISO were not altered by the previous administration of the drug. In contrast, a similar comparison for the phenytoin group data revealed a number of differences between the two doses, which could therefore be attributed to the phenytoin administration.

The half-life of MISO was considerably shortened (mean reduction 31 ± 9%, s.d., $P < 0·001$) (Table I). This effect was seen for all 6 patients, but was more marked in those with longer initial half-lives. The area under the curve (AUC) of plasma MISO concentration against time was also reduced by a similar amount (mean reduction 29 ± 14%, s.d., $0·01 > P > 0·001$) (Table I). In contrast, the peak plasma concentration for the metabolite DEMIS was markedly increased (mean increase 75 ± 16%, s.d., $P < 0·001$) (Table I) as was the AUC (69 ± 49%, s.d., $0·05 > P > 0·02$) (data not shown). However, neither the peak plasma MISO concentration (Table I) nor the plasma concentration 4 h after administration of the drug (time of irradiation) (Table II) was affected by phenytoin ($P > 0·1$). In 4/6 patients the AUC for total plasma 2-nitroimidazole (MISO + DEMIS) was reduced after phenytoin administration, but this was not significant for the whole group ($P > 0·1$).

TABLE II.—*Plasma misonidazole concentrations 4 h after drug administration (mean ± s.d., n = 6 per group)*

| | Plasma misonidazole (mM) at 4 h | |
|---|---|---|
| | 1st dose | 2nd dose |
| Control | 0·40 ± 0·08 | 0·41 ± 0·10 |
| Phenytoin | 0·58 ± 0·25 | 0·53 ± 0·15 |

The above data clearly indicate a marked effect of a short course of phenytoin on the pharmacokinetics of MISO. The shortened half-life and reduced AUC for plasma MISO were associated with a concomitant increase in the concentrations of oxidative metabolite DEMIS. These effects are similar to our recent observations with both phenytoin and phenobarbitone in mice and dogs (Workman, 1979; White & Workman, 1980).

Phenytoin is known to increase the rate of elimination of many drugs, in experimental animals and in man, by acting as a potent inducer of hepatic microsomal drug-metabolizing enzymes (Conney, 1967; Pirttiaho *et al.*, 1978). Our data indicate that such induction increases the rate of oxidative demethylation of MISO to DEMIS.

Clinical doses of MISO are limited by its neurotoxicity, particularly peripheral neuropathy, and a total dose not exceeding 12 $g/m^2$ is now recommended (Dische *et al.*, 1977; Urtasun *et al.*, 1978). Moreover, there is evidence that the incidence of neuropathy is related to the plasma AUC for MISO, and dose adjustment on the basis of plasma pharmacokinetics has been advocated (Saunders *et al.*, 1978). It therefore appears possible that patients exposed to potent hepatic enzyme-inducing agents, such as phenytoin and phenobarbitone, may be protected to some extent from neurotoxicity by rapid drug clearance. It has not yet been determined, however, whether there is any correlation between phenytoin or phenobarbitone administration and the clinical incidence or severity of MISO neurotoxicity. A study on these lines is in progress.

We should also consider the possible effects of phenytoin on the therapeutic action of MISO. The degree of radiosensitization is a function of tumour MISO concentration during irradiation (McNally *et al.*, 1978) and this is closely related to plasma concentration (Dische *et al.*, 1977; Ash *et al.*, 1979). Current clinical practice is to irradiate 4 h after MISO administration, and we have shown that neither the peak plasma MISO concentration nor the 4 h concentration is affected by phenytoin. In addition, we have previously shown that peak tumour MISO concentrations are not altered in mice pretreated with phenytoin or phenobarbitone (Workman, 1979). We therefore feel it is unlikely that phenytoin administration would affect the radiosensitization of tumour cells by MISO. MISO also exhibits selective cytotoxicity towards hypoxic cells (Hall & Roizin-Towle, 1975; Stratford & Adams, 1978). However, its clinical significance and the effects of enzyme induction on that are unknown.

In conclusion, phenytoin, and possibly other potent liver enzyme-inducing agents such as phenobarbitone, may reduce the incidence of dose-limiting neurotoxicity of MISO without compromising the direct tumour radiosensitization by the drug. The possible effects on the drug's antitumour cytotoxicity are uncertain.

We thank Mr L. S. Freedman for statistical advice; Dr Nancy C. Smith and Mrs Jane Donaldson for excellent technical assistance; and Dr C. E. Smithen and Dr I. Lenox-Smith of Roche Products Limited for supplies of misonidazole and other nitroimidazoles.

### REFERENCES

Ash, D. V., Smith, M. R. & Bugden, R. D. (1979) Distribution of misonidazole in human tumours and normal tissues. *Br. J. Cancer*, **39**, 503.

Conney, A. H. (1967) Pharmacological implications of microsomal enzyme induction. *Pharmacol. Rev.*, **19**, 317.

Dische, S., Saunders, M. I., Lee, M. E., Adams, G. E. & Flockhart, I. R. (1977) Clinical testing of the radiosensitiser Ro 07–0582: experience with multiple doses. *Br. J. Cancer*, **35**, 567.

Hall, E. J. & Roizin-Towle, L. (1975) Hypoxic sensitisers: Radiobiological studies at the cellular level. *Radiology*, **117**, 453.

McNally, N. J., Denekamp, J., Sheldon, P. W., Flockhart, I. R. & Stewart, F. A. (1978) Radiosensitisation by misonidazole (Ro 07–0582). The importance of timing and tumour concentration of sensitiser. *Radiat. Res.*, **73**, 568.

Pirttiaho, H. I., Sotaniemi, E. A., Ahokas, J. T. & Pitkänen, U. (1978) Liver size and indices of drug metabolism in epileptics. *Br. J. Clin. Pharmacol.*, **6**, 273.

Saunders, M. I., Dische, S., Anderson, P. & Flockhart, I. R. (1978) The neurotoxicity of misonidazole and its relationship to dose, half-life and concentration in the serum. *Br. J. Cancer*, **37** (Suppl. III), 268.

Stratford, I. J. & Adams, G. E. (1978) The toxicity of the radiosensitiser misonidazole towards hypoxic cells *in vitro*: A model for mouse and man. *Br. J. Radiol.*, **51**, 745.

Urtasun, R. C., Chapman, J. D., Feldstein, M. L. & 6 others (1978) Peripheral neuropathy related to misonidazole: incidence and pathology. *Br. J. Cancer*, **37** (Suppl. III), 271.

White, R. A. S. & Workman, P. (1980). Phenytoin sodium-induced alterations in the pharmacokinetics of misonidazole in the dog. *Cancer Treatment Rep.* (in press).

Workman, P. (1979) Effects of pretreatment with phenobarbitone and phenytoin on the pharmacokinetics and toxicity of misonidazole in mice. *Br. J. Cancer*, **40**, 335.

Workman, P., Little, C. J., Marten, T. R. & 4 others (1978*a*) Estimation of the hypoxic cell-sensitiser misonidazole and its O-demethylated metabolite in biological materials by reversed-phase high-performance liquid chromatography. *J. Chromatogr.*, **145**, 507.

Workman, P., Wiltshire, C. R., Plowman, P. N. & Bleehen, N. M. (1978*b*) Monitoring salivary misonidazole in man: a possible alternative to plasma monitoring. *Br. J. Cancer*, **38**, 709.